# Ambivalent Sexism and Religion: Connected Through Values

**DOI:** 10.1007/s11199-014-0379-3

**Published:** 2014-05-28

**Authors:** Małgorzata Mikołajczak, Janina Pietrzak

**Affiliations:** Faculty of Psychology, University of Warsaw, Stawki 5/7, 00-183 Warsaw, Poland

**Keywords:** Religiosity, Ambivalent sexism, Benevolent sexism, Values, Conservatism

## Abstract

Sexist attitudes do not exist in a limbo; they are embedded in larger belief systems associated with specific hierarchies of values. In particular, manifestations of benevolent sexism (Glick and Fiske 1996, 1997, 2001) can be perceived as a social boon, not a social ill, both because they are experienced as positive, and because they reward behaviors that maintain social stability. One of the strongest social institutions that create and justify specific hierarchies of values is religion. In this paper, we examine how the values inherent in religious beliefs (perhaps inadvertently) propagate an unequal status quo between men and women through endorsement of ideologies linked to benevolent sexism. In a survey with a convenience sample of train passengers in Southern and Eastern Poland (*N* = 180), we investigated the relationship between Catholic religiosity and sexist attitudes. In line with previous findings (Gaunt 2012; Glick et al. 2002a; Taşdemir and Sakallı-Uğurlu 2010), results suggest that religiosity can be linked to endorsement of benevolent sexism. This relationship was mediated in our study by the values of conservatism and openness to change (Schwartz 1992): religious individuals appear to value the societal status quo, tradition, and conformity, which leads them to perceive women through the lens of traditional social roles. Adhering to the teachings of a religion that promotes family values in general seems to have as its byproduct an espousal of prejudicial attitudes toward specific members of the family.

## Introduction

To illuminate how women’s roles are circumscribed by both positive and negative attitudes, Glick and Fiske (1996, 1997, 2001) introduced Ambivalent Sexism Theory, according to which perceiving women as possessing both positive (e.g., warm, caring) and negative (e.g., manipulative, unreliable) characteristics simultaneously justifies and propagates inequalities between men and women (e.g., Cikara et al. 2009; Connelly and Heesacker 2012; Jost and Kay 2005; Sibley et al. 2007). These beliefs about the essential characteristics of women, and the essential differences between men and women, making each more or less adequate to fill particular roles in society, appear to be relatively universal (Fortin 2005; Harris 1991; Hofstede 1980). The ambivalence this division entails for both men and women has been observed in a number of countries (Glick et al. 2000, 2004).

Gender role expectations are also influenced by cultural factors, including religion (Burn and Busso 2005 [studied in the United States]; Gaunt 2012 [in Israel]; Glick et al. 2002a [in Spain]; Taşdemir and Sakallı-Uğurlu 2010 [in Turkey]). The link between religion and sexism might be direct—religious authorities might explicitly teach stereotypical views of the genders. However, it is likely that there is also a more indirect connection, the result of broader values incorporated into religious teachings. A number of studies have demonstrated how religiosity is connected to a specific hierarchy of values, ascribing great weight to guarantees of stability and certainty (reviewed in, e.g., Roccas 2005; Saroglou and Cohen 2011). Such attachment to tradition and uncertainty avoidance can affect how one responds to norm adherence and norm violation and, more specifically, to individuals who comply with or violate social norms.

In this paper, we demonstrate how religion can promote benevolent sexism by way of emphasizing traditional values. Measuring religiosity, ambivalent sexism (Glick and Fiske 1996) and values (Schwartz et al. 2001) among Polish men and women, we show an indirect pathway from embracing socially favored religious teachings (Schwartz et al. 1997), to socially undesirable prejudice, sexism. Exploring this indirect pathway illuminates how the seemingly prosocial aspects of religious discourse—laudatory, appreciative commentary about women’s place in society—promote a broader belief system that stabilizes the unequal status quo. While our work focuses on the links between Catholic religiosity and sexism in Poland, it is plausible that such connections are to be found in other religions and cultures; we discuss this further below. Uncovering these indirect, likely unconscious links can serve as a basis for understanding implicit, deep-rooted barriers to attitude and behavioral change.

### Ambivalent Sexism Theory

Ambivalent Sexism Theory posits two forms of sexism that differ in their subjective experience and reception but sprout from the same roots. A lay understanding of sexism is likely to revolve around hostile, adversarial feelings toward women—what Glick and Fiske (1996) termed *hostile sexism*. Hostile attitudes toward women are reflected in the notions that women demand special favors (“When women lose to men in a fair competition, they typically complain about being discriminated against”) and vie for power over men (“Once a woman gets a man to commit to her, she usually tries to put him on a tight leash”).

A mirror image of hostile sexism, that is, a supportive and kindly view toward woman, was termed *benevolent sexism*. Benevolent attitudes toward women are reflected in the notions that women are morally superior to men (“Many women have a quality of purity that few men possess”) and require male protection (“Women should be cherished and protected by men”). Although benevolent sexism can be experienced by both the perceiver and the target as positive, its roots run to the same gender ideology as hostile sexism, and its consequences are often negative. For example, stereotyping women as more righteous and virtuous than men imposes on them greater responsibility for moral conduct in the sexual sphere (Abrams et al. 2003 [studied in England]).

What might seem to be irreconcilable ambivalence toward women (women are power-hungry and manipulative, but they are also morally pure) in practice appears to manifest as two unipolar attitudes directed at different types of women (Glick et al. 1997 [United States]). Women who adhere to more traditional roles are valued and rewarded, while women who attempt to break out of the gender hierarchy are treated with hostility and suspicion. Benevolent sexists believe that deviations from a prescriptive set of norms should be met not with protection but with punishment (Glick et al. 2002b [in Turkey and Brazil]; Whatley 2005 [in the United States]; Yamawaki et al. 2007 [in the United States]).

Religion cultivates such reward-punishment contingencies. For example, Burris and Jackson (1999) have shown that traditional denominations contribute to the acceptance of violence in romantic relationships. In their study, religious individuals in Canada kept the side of the victim of abuse only when she conformed to religiously inscribed values, and not when she contradicted them (e.g., by being a lesbian). The victim’s behavior was considered a reasonable justification for otherwise socially disapproved attitudes—sympathy for the perpetrator and liberal judgments of his behavior.

### The Catholic Church and Traditional Gender Roles

In its official teachings, the Catholic Church clearly propagates the idea of men’s (e.g., dominance, responsibility) and women’s (e.g., cordiality, humility) disparate traits and naturally resulting distinct roles (particularly, women as mothers who self-sacrifice and care for others; Benedict XVI 2005; John Paul II 1988, 1995; Ratzinger and Amato 2004). By placing emphasis on uniquely female versus male traits and the interdependence between the genders, justifying the division into traditional roles, Catholicism may contribute to the popularity of favorable attitudes that are directed only towards women who fit their “God-given” roles such as that of a mother. Such attitudes, as has been shown in other research, *ipso facto* yield negative consequences, preserving social inequalities between the genders (e.g., Cuddy et al. 2004 [United States]). Indeed, a positive relationship between Catholic religiosity and benevolent sexism has been found in a random telephone sample of Spanish adults (Glick et al. 2002a).

Though differences within Christianity do exist—for example, although some Christian denominations allow women to be priests (e.g., Protestantism), this is not the case in Catholicism—the ideological narratives that underlie Catholicism could be embedded in the scripts of other Christian denominations. Similarly, despite discrepancies between Islam, Judaism and Christianity, both Jewish tradition and Islamic scripts concerning gender resemble those of the Bible. Few studies have looked at evidence for ambivalent sexism within different denominations and religions; however, the few that have showed patterns of results similar to those found for the Catholicism. In a sample of Evangelical Christians in the United States (Maltby et al. 2010), men’s level of protective paternalism was positively linked to the measure of religious orthodoxy. Similarly, in a sample of American students who self-identified as Christians (including Catholics, Orthodox, and various Protestant groups, Burn and Busso 2005), benevolent sexism of both men and women correlated positively with level of religiosity. A study conducted in predominantly Muslim Turkey replicated findings from Christian samples, showing a positive link between religiosity and benevolent sexism among university students (Taşdemir and Sakallı-Uğurlu 2010). Gaunt (2012) found the same patterns in a large convenience sample of adult Israeli Jews. Thus, it appears that other denominations and religions pass on teachings if not of identical content, at least of similar overtone (e.g., Benson and Stangroom 2009; Stover and Hope 1984). We believe that these beliefs about and attitudes towards men and women, and the relations between them, are related to more global, encompassing belief constructs: values.

### Traditional/ Religious Values as Frameworks for Attitudes

The broad pattern of values promoted by religion can determine how adherents perceive and construe issues even beyond the teachings of a given church. The influence of religion on phenomena that are not strictly religious has been a topic of discussion and empirical research (e.g., Saroglou and Cohen 2011). For instance, Protestantism promotes economic values linked to capitalism, on both an individual and a national level (Hayward and Kemmelmeier 2011), and Christianity and Buddhism have an impact on affective states valued by their respective practitioners (Tsai et al. 2007 [United States]).

Values are most commonly defined in contemporary psychological literature after Schwartz (1992) as “desirable goals, varying in importance, that serve as guiding principles in people’s lives” (p. 89). The key element distinguishing specific values is the type of motivational goal they serve. Schwartz (1992) distinguished ten value types, which can be arranged on two dimensions: *openness to change* (self-direction, stimulation, hedonism) versus *conservatism* (tradition, conformity, security); and *self-transcendence* (benevolence, universalism) versus *self-enhancement* (power, achievement). For example, a person who is guided by universalism will value all of humanity and seek to promote social justice. This value might translate into low levels of prejudice against a variety of outgroups. A person who values security, on the other hand, might espouse positive prejudice toward groups that are high in the social hierarchy, and negative prejudice toward groups that have low status. This way, we propose, her specific beliefs can buttress her more general preference for a stable social order (cf. Jost et al. 2004; Kay et al. 2005).

Religions provide a fairly consistent way of organizing and prioritizing values, such that religiosity has been consistently shown to be linked to particular values in many countries (Saroglou et al. 2004; Schwartz and Huismans 1995). Moreover, societies attribute importance to particular values in line with the dominant religion (Inglehart 2000). A meta-analytical review of studies using Schwartz’s model by Saroglou and colleagues (2004) included 21 samples from 15 countries of different religious denominations—Christians, Jews and Muslims. Regardless of denomination, religious individuals assigned high ranks to values related to self-transcendence (i.e., benevolence and universalism), preservation of the social status quo (tradition, conformity), and protection against uncertainty (security); and relatively low ranks to values promoting hedonism and intellectual or emotional openness to change (self-direction). These results can be interpreted as indicators of religion’s pro-social foundations. Religious individuals support the broader social good over the individual good; the known over the unknown; social norms over individual decision-making. These are values that allow for the creation and maintenance of structure and order in society. They are also values that can lead to selective intolerance of those who threaten or disrupt the social order (e.g., Altemeyer and Hunsberger 1992 [in Canada]; Duck and Hunsberger 1999 [Canada]; Rowatt et al. 2009 [United States]).

Feather (2004) has proposed that values guide information processing by sensitizing an individual to information relevant to her values, which directs perceptions and behavior. Because values have the power to influence the process of adopting certain attitudes, people may adhere to ideologies that are coherent with the values they endorse (Katz and Hass 1988 [United States]). Ambivalent attitudes toward women may therefore be a reflection of specific values. Indeed, while both hostile and benevolent sexism can be predicted by higher endorsement of power values and lower endorsement of self-transcendence (benevolence and universalism), only benevolent sexism correlates positively with tradition and negatively with self-direction (Feather 2004; Feather and McKee 2012 [Australia]).

### Why is There a Link Between Religious Values and Sexism?

A direct link between religiosity and sexism has been shown (e.g., Morgan 1987); the process by which religiosity leads to sexism remains under-researched. A recent analysis using data from the World Values Survey looked at the relationships between religion and gender attitudes in general (Seguino 2011). Regression analyses showed a significant impact of religiosity, assessed in terms of both subjective importance and participation, on gender attitudes. Furthermore, these effects were explained by political attitudes rooted in religious ideology, which shaped views on issues that had specific economic consequences, such as labor force participation or maternity leave policies. This was true irrespective of religion. This hints at the possibility of underlying values driving both kinds of attitudes.

Incorporating the distinction between hostile and benevolent attitudes into such analyses further illuminates the mechanisms through which religion might influence gender attitudes. Data collected in predominantly Catholic Spain show that higher levels of religiosity were associated with higher levels of benevolent sexism, while being unrelated to hostile sexism (Glick et al. 2002a). Both intrinsic and extrinsic religiosity (Allport and Ross 1967), as well as scriptural literalism, correlate positively with benevolent sexism (and are unrelated to hostile sexism; Burn and Busso 2005 [United States]). Glick and colleagues (2002a) speculate that religiosity fosters ambivalent attitudes, chiefly those related to benevolent sexism, but the process by which this happens, and the direction of the influence, are unclear.

One such process might link sexism to broader values communicated by religious teachings. Specifically, drawing on previous evidence (Feather 2004; Saroglou et al. 2004), it might be conjectured that religiosity will be related to sexism indirectly, via conservatism. To the extent that religious individuals attach relatively high importance to tradition and conformity—opting for the preservation of the status quo rather than for social change in general—they might also be more likely to endorse traditional beliefs ingrained in benevolent sexism. At the same time, the pro-social values of universalism and benevolence conveyed through religion might indirectly contribute to a weaker endorsement of sexism. To the extent that religious individuals attach relatively high importance to the good of their proverbial neighbors and justice in general, they might also be less likely to endorse sexist beliefs.

### Gender Equality and Catholic Religiosity in Poland

Poland offers a relevant empirical context for investigating mechanisms related to Catholic religiosity. The Catholic Church is a key shaper of values and attitudes in Polish society, on both the individual and the societal levels. As the dominant denomination, Catholicism is inscribed in the socio-cultural landscape and constitutes an important ingredient in Poles’ national identity (Heinen and Portet 2009). According to representative surveys, 93 % of Poles declare themselves to be Catholic, of whom 67 % rate themselves as religious (Centrum Badania Opinii Społecznej 2012). Almost half (48 %) of Polish citizens claim that public social life should be based on values proclaimed by religion (Centrum Badania Opinii Społecznej 2007). And, indeed, it is. The influence of the Catholic Church on the political system in Poland is strong (Chełstowska et al. 2013). This influence springs partly from a boomerang response to Communist rule, during which the Church was absent from official public life. After the political transformation in Poland, any opposition to the Church’s postulates about public policy were taken as an indication of support for the previous “totalitarian” regime. Thus, the Church’s input into the writing of the Polish Constitution, as well as later laws, has been much greater than the declarative separation of Church and State would suggest.

Many Poles “continue to be believers first, and citizens second” (Marsh 2009, p. 33). This influences the kinds of gender discrimination issues that come up for public debate. Much of the focus of feminist and women’s organizations in Poland has been on reproductive rights, while less overt forms of discrimination are dismissed or trivialized (Marsh 2009). This can partially be explained by the relatively strong presence of women in the workforce. During the Communist rule, all citizens were expected to participate actively in the labor market. Although the fall of Communism was officially announced in Poland in 1989, some effects of its policies endure: women still expect and are expected to work outside the home. The decades of women’s labor participation mean that both men and women are accustomed to idea of women playing both professional and domestic roles. Currently, Poland ranks 54th worldwide in terms of women in parliament, with 24 % of the lower house and 13 % of the Senate (Inter-Parliamentary Union 2013). The gender inequality index is 0.14, giving Poland a rank of 24th worldwide (United Nations Development Programme 2013). These (relatively) positive rankings belie gender attitudes that are still quite traditional on the homefront, even among young adults. While in national surveys 46 % of couples declare a preference for a partnership model of marriage, women are still vastly more likely to have full responsibility for household chores such as laundry, meal preparation, and cleaning (Centrum Badania Opinii Społecznej 2013b). Research with student samples in Poland has indicated stronger hostile- and benevolent-sexist attitudes than those found among samples in South Africa and the United Kingdom (Zawisza et al. 2013) and in the United States (Forbes et al. 2004).

### Overview of the Present Study

Because religion has the power to establish value hierarchies, and the acceptance of distinct gender roles can be linked to religiosity (Burn and Busso 2005), Catholicism and the level of religiosity of its followers may contribute to the popularity of specific forms of sexism in predominantly Catholic countries. A study was designed to investigate these relationships showing that Catholic church attendance can be linked to benevolent sexist attitudes through traditional values, taking into account the effects of gender and education.

In the study, we tested hypotheses linking Catholic religiosity, benevolently sexist attitudes, and personal values.

Based on previous work by Saroglou and colleagues (2004), Glick and colleagues (2002a, b), as well as Schwartz and Huismans (1995), we made the following two predictions, one concerning a direct effect, the other concerning an indirect effect:

1. Greater Catholic religiosity will be associated with higher benevolent sexism among both women and men.
2. The link between Catholic religiosity and benevolent sexism will be mediated by values among both women and men. Specifically, we predict:
3. a positive indirect effect via values of Conservatism;
4. a negative indirect effect via values of Self-transcendence.

To test these hypotheses, we used regression analysis (Hypothesis 1) and mediation analysis (Hypothesis 2).

Being aware of the potential interplay of religiosity, sexism and values with participant’s age and education, we included these socio-demographic variables as covariates in all analyses. Previous studies have shown that even within a relatively homogenous cultural and religious context, sexist attitudes are likely to vary with education and age (Gaunt 2012; Glick et al. 2002a). Similarly, people tend to value security more and hedonism less with age; the more educated they are, the more they value self-direction and the less tradition (Schwartz 1992). Moreover, education has been shown to interact with gender: among students, gender differences in values were weaker than in non-student samples of comparable age (Schwartz and Rubel 2005). However, having no theoretical or empirical support for hypotheses concerning these variables, we made no specific predictions concerning the influence of demographic characteristics on the studied relationships per se. For the same reason, investigations into links between hostile sexism and other variables are, at this time, purely exploratory.

## Method

### Participants and Procedure

Completed questionnaires were gathered from 189 respondents. Participants were recruited from among passengers of long-distance trains running on two routes in the Southern (Warsaw-Cracow) and Eastern (Warsaw-Lublin) parts of Poland. The analyzed sample consisted of 180 participants: 159 respondents who declared themselves as Catholics, and 21 respondents who indicated no religious affiliation. To reduce ambiguity in interpreting our religiosity variable, we excluded six respondents who declared religious denominations other than Catholicism (1 Orthodox, 3 Protestant, 1 Judaic, and 1 Buddhist) and three who did not respond to the question. Our final sample was less religious than a representative sample of Poles would be: it consisted of 12 % non-believers, 22 % non-practitioners, 41 % irregular practitioners and 25 % regular practitioners. The representative percentages would be 5 %, 7 %, 37 %, and 51 %, respectively (Centrum Badania Opinii Społecznej 2013a).

The sample consisted of 104 women and 76 men. The mean age of participants (18–77, *M* = 33.49, *SD* = 13.76) was close to the median age of a statistical Pole (*Mdn* = 38.4), but our sample was better educated than a representative sample of Poles of a similar age would be: 51 % of our participants had a higher education, 16 % incomplete higher, 9 % postsecondary, and 23 % had completed secondary or lower education. A representative sample would have 33 %, n/a, 3 %, and 64 %, respectively (Główny Urząd Statystyczny 2013). Table 1 shows the demographic characteristics by gender in the final sample. No gender differences were found for education, age or religiosity.

**Table 1:** Demographic characteristics of the participants

	Women (*n =* 104)		Men (*n* = 76)	
	*n*	%	*n*	%
Education				
Secondary or lower	27	26.0	15	20.0
Postsecondary	8	7.7	8	10.7
Incomplete higher	14	13.5	15	20.0
Higher	55	52.9	37	49.3
Religiosity				
Non-believers	8	7.8	13	17.3
Non-practitioners	20	19.6	19	25.3
Irregular practitioners	47	46.1	26	34.7
Regular practitioners	27	26.5	17	22.7
Age				
Mean	34.22		32.49	
SD	13.31		14.38	
Range	18–67		18–77	

*Note.* There were no significant differences between men and women in the reported variables

Participants were approached in their compartments during an approximately 3-h train ride by a female undergraduate research assistant who introduced the study as an attitude survey. All responses were anonymous. It took participants approximately 15 min to complete the survey.

### Measures

#### Ambivalent Sexism

We used a back-translated Polish version of the Ambivalent Sexism Inventory (ASI; Glick and Fiske 1996). Following Glick et al.’s (2000) suggestion, we used non-reversed wording of all items. The ASI consists of 22 statements (see Appendix for English items and their Polish wording), half of them comprising the hostile subscale (α = .89, *M* = 2.63, *SD* = 1.06 in our sample) and half the benevolent subscale (α = .81, *M* = 2.97, *SD* = 0.98). Participants rated each statement on a six-point Likert-type scale, from 0 (*strongly disagree*) to 5 (*strongly agree*).

#### Values

We used an abbreviated version of the Schwartz et al.’s (2001) Portrait Value Questionnaire (PVQ). In the PVQ, 21 descriptions of people matching the respondent’s gender are presented (e.g., “It’s very important to [him/her] to help the people around [him/her]. [He/She] wants to care for other people”) and the respondent’s task is to assess his or her subjective similarity with each. The greater the similarity (indicated on a six-point Likert-type scale from 1 to 6), the more important the value that they represent to the respondent.

Following Schwartz (2003), we centered each person’s responses on his or her own mean in order to eliminate individual differences in use of the response scale. Cronbach’s alphas for the particular scales were: .67 for conservatism (*M* = −0.16, *SD* = 0.78), .73 for openness to change (*M* = −0.06, *SD* = 0.76), .72 for self-enhancement (*M* = −0.44, *SD* = 0.82), and .65 for self-transcendence (*M* = 0.64, *SD* = 0.66). Because the values were highly correlated, we performed a Principal Components Analysis (with the varimax rotation), which revealed two factors (eigenvalues >1) that corresponded with Schwartz’s distinction of two bipolar dimensions: conservatism vs. openness to change; and self-transcendence vs. self-enhancement. For the sake of simplicity, these two dimensions are hereafter called Conservatism and Self-transcendence. Composite value measures were computed by subtracting the opposing value scores, such that higher scores indicate greater Conservatism (*M* = 0.01, *SD* = 1.46) and greater Self-transcendence (*M* = 1.09, *SD* = 1.38).

#### Religiosity

Religiosity was assessed with three items. The first asked whether the participant was Catholic (*yes/no*). If respondents were not Catholic, they were asked to indicate their religion, if any (*open-ended*). This allowed us to eliminate individuals of other faiths. The third question was a one-item ordinal measure of the frequency of church attendance (*regular/ irregular/ none*). Because approximately 95 % of Poles come from a Catholic background (Centrum Badania Opinii Społecznej 2009), the relevant distinction in this context is not between different denominations but the strength of association with Catholicism. Therefore, following Glick et al. (2002a), we assessed Catholic religiosity by collapsing responses to the questions about religious denomination with frequency of church attendance. Based on these questions, we composed an ordinal measure with four categories of (Catholic) religiosity: nonbelievers (Catholic*: no,* other denomination: *no,* church attendance: *none*), non-practitioners (Catholic*: yes,* church attendance: *none*), irregular practitioners (Catholic*: yes,* church attendance: *irregular*), and regular practitioners (Catholic*: yes,* church attendance: *regular*).

As Tarakeshwar et al. (2003) noted in their meta-analysis, frequency of attendance is a global indicator typically used in studies dealing with religion. In previous research, this measure proved to be satisfactory when predicting gender division of labor (Sanchez and Hall 1999) and benevolent sexism (Glick et al. 2002a).

#### Demographics

Participants answered questions concerning their gender, age, and educational level. Categories used are shown in Table 1.

## Results

### Gender Differences in Values and Sexism

Gender differences in sexism and values were computed in a MANCOVA controlling for participants’ age and educational level (see Table 2). Men scored higher than women both on hostile, *F*(1,176) = 24.63, *p* < .001 and benevolent sexism, *F*(1,176) = 6.51, *p* < .05, and these effects were stronger for hostile attitudes (partial η^2^ = .13) than for benevolent attitudes (η^2^ = .04). We found no significant gender differences in mean scores for value dimensions.

**Table 2:** Means and standard deviations for all continuous variables by participant gender

	Women		Men		Multivariate analysis of covariance (MANCOVA)	
Variables	*M*	*SD*	*M*	*SD*	*F* (1,176)	Partial η^2^
Hostile sexism	2.30_a_	1.00	3.06_b_	.98	24.63***	.13
Benevolent sexism	2.81_a_	1.00	3.15_b_	.90	6.51*	.04
Conservatism	−.12	.84	−.25	.65	.55	.00
Openness to change	−.12	.83	.07	.59	2.14	.01
Self-transcendence	.68	.71	.55	.56	.63	.00
Self-enhancement	−.49	.86	−.38	.76	.38	.00

*Note.* Scale endpoints for the two sexism subscales were 0 and 5. For each person, value scores on a 1–6 scale were centered around his/her mean, thus the score could range from −5 to 5. Differences between male and female participants were tested with a MANCOVA (controlling for participant’s age and educational level), which revealed a significant main effect of gender, Wilks λ = .85, *F* (6, 167) = 4.97, *p* < .001, partial η^2^ = .15. Means that are significantly different between men and women are denoted with different subscripts within rows

*p < .05, ***p < .001

### Religiosity and Sexism

Correlations between all continuous study variables were computed separately for women and men (see Table 3). Following Glick and Fiske (1996), partial correlations were used to test the link between religiosity and benevolence while controlling for hostility and vice versa.

**Table 3:** Correlations between ASI, values and religiosity

Variable	1	2	3	4	5	6	7
1.Hostile sexism	–	.58**	.01	−.19†	.08	−.08	−.09
2.Benevolent sexism	.32**	–	.18†	.04	.27**	.01	−.19†
3.Conservatism	.09	.23†	–	.48**	.24*	.54***	−.10
4.Self-transcendence	−.03	.04	.19	–	.07	.49***	.01
5.Religiosity	.05	.10	.43***	.05	–	−.03	−.24*
6.Age	.05	−.01	.25*	.29*	.04	–	.07
7.Education	−.26*	.00	−.01	−.10	.03	−.02	–

*Note*. Intercorrelations among men (*n* = 76) are presented below the diagonal, and intercorrelations among women (*n* = 104) are presented above the diagonal. Higher values on all measures indicate higher construct level. For correlations with benevolent sexism and hostile sexism, partial coefficients controlling for positive link between the two constructs are shown

† *p* < .10, * *p* < .05, ** *p* < .05

#### Direct Effects

To test the effect of Catholic religiosity on sexism (Hypothesis 1), we performed regression analyses separately for men and women (see Table 4). To control for the positive correlations between hostile and benevolent sexism, the complementary form of sexism was entered in the first step as a control variable, together with age and education. Catholic religiosity was then entered in the second step to assess its unique effect on sexism above and beyond demographic variables. All variables were assessed for possible multicollinearity using tolerance and the variance inflation factor (VIF). Tolerance values of .829 to .996 and VIF values of 1.003 to 1.206 indicated that multicollinearity was not an issue in either of the equations. Although all models reached significance, religiosity was a significant predictor of benevolent sexism only among women, β = .20, *p <* .05, not men, β = .09, *ns*. Thus, Hypothesis 1 was only partially supported.

**Table 4:** Regression equations predicting sexism from religiosity and socio-demographic variables

		Women		Men	
		HS	BS	HS	BS
Step 1	(BS/HS)^a^	.54***	.53***	.35**	.38**
	Age	−.06	.01	.04	−.01
	Education	−.08	−.16†	−.24*	.00
	R^2^	.33***	.34***	.20***	.14*
	F	15.74***	16.87***	5.84***	3.83*
	df	3, 100		3, 73	
Step 2	(BS/HS)^a^	.52	.48***	.34**	.37**
	Age	−.06	.01	.04	−.01
	Education	−.07	−.12	−.24*	−.01
	Religiosity	.05	.20*	.06	.10
	ΔR^2^	.01	.04*	.01	.01
	F	11.82***	14.65***	4.41**	3.05*
	df	4, 100		4, 73	

*Note.* Standardized beta coefficients are reported

*HS* hostile sexism, *BS* benevolent sexism

^a^HS was entered as a control variable to the regression equations of BS, BS was entered as a control variable to the regression equations of HS

† *p* < .10 *p < .05. **p < .01. ***p < .001

For both genders, religiosity was unrelated to hostile sexism (β = .05, *ns*, and β = .06, *ns*). Moreover, age was not a significant predictor of sexism in either of the equations. Higher educational attainment predicted lower hostile sexism (β = −.24, *p* <. 05) among men, but not women (β = −.07, *ns*) and was unrelated to benevolent sexism.

#### Indirect Effects Through Values

In order to test Hypotheses 2a and 2b, concerning indirect effects of Catholic religiosity on benevolent sexism via the values of Conservatism and Self-transcendence, we conducted mediation analyses controlling for hostile sexism, age and education (the pattern of results did not change when these variables were not controlled for).

We followed the procedure described by Preacher and Hayes (2008) to test for indirect and direct effects in a multiple mediator model. We used the PROCESS macro (Model 4, Hayes 2013) and requested 10,000 bootstrap samples.

**Fig. 1:**
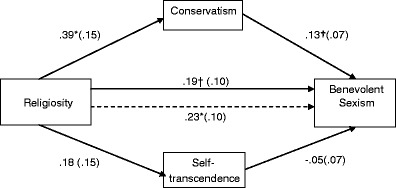
Mediation effects of religiosity on benevolent sexism through endorsed values among women. *Note:* †*p* < .10, *p < .05. Entries are unstandardized regression coefficients with standard errors in parentheses. The dotted line indicates the path for simple regression (not controlling for mediators)

**Fig. 2:**
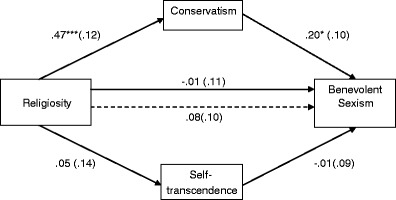
Mediation effects of religiosity on benevolent sexism through endorsed values among men. *Note:* †*p* < .10, *p < .05, ***p < .001. Entries are unstandardized regression coefficients with standard errors in parentheses. The dotted line indicates the path for simple regression (not controlling for mediators)

Among women (see Fig. 1), the indirect effect of religiosity on benevolent sexism via values was positive and significant for Conservatism, *B* = .05, *SE* = .04. The indirect effect had a bootstrap 95 % bias corrected confidence interval of .005 to .135. For Self-transcendence, the corresponding interval was −.071 to .010, indicating that the effect was non-significant. Similarly, among men (see Fig. 2) the indirect effect of religiosity on benevolent sexism via Conservatism was positive and significant, *B* = .10, *SE* = .04, 95 % CI [.024, .207] and was insignificant via Self-transcendence, 95 % CI [−.037, .021]. Thus, Hypothesis 2a was supported and Hypothesis 2b was rejected in both genders.

To address possible alternative causality, we checked the significance of the indirect effects in a reversed mediation model, with value dimensions as independent variables and Catholic religiosity as a mediator. We used the MEDIATE macro (Hayes 2013) and requested 10,000 bootstrap samples (Hayes and Preacher 2013). For both women and men, we obtained non-significant indirect effects for each of the values. Similarly, in models with hostile sexism as a dependent variable, none of the indirect effects reached significance.

## Discussion

In this study, we examined whether religiosity could contribute both directly and indirectly to the popularity of ambivalent sexism. Results partially confirmed previous findings (Burn and Busso 2005; Gaunt 2012; Glick et al. 2002a; Maltby et al. 2010; Taşdemir and Sakallı-Uğurlu 2010): among women (but not men) religiosity proved to reinforce benevolent sexism, and was unrelated to hostile sexism.

Extending previous findings, we showed that among both genders the relationship between religiosity and benevolent sexism was mediated by attachment to Conservatism, values that can be likened to traditional world-views—respect for tradition, submission to societal norms, support for the status quo, and intellectual and emotional interdependence.

These results support the argument (e.g., Glick et al. 2002a) that benevolent sexism, as long as it is considered a positive attitude, is propagated by legitimate social institutions, such as the Catholic Church. We extend previous findings by exploring the mechanisms underlying this connection. Religiosity shapes sexist attitudes not only directly, but also indirectly, by referring to traditional norms and values and through the conventionalism of gender roles it advocates. It seems that church attendance, linked positively to valuing conformity, tradition, and security over openness to change, leads Catholics to an appreciation of women in their unique role as delicate, moral caretaker. Even if their proclaimed content takes only the benevolent form, these ideologies sustain gender inequalities on a societal level.

Our hypotheses were not fully supported, however, nor do our results fully replicate previous findings. Religion was not directly linked to benevolent sexism among men in our sample. This is inconsistent with previous research using similar measures of religiosity (Gaunt 2012; Glick et al. 2002a). It is plausible that the gender differences in links between different forms of religiosity and subcomponents of benevolent sexism—particularly protective paternalism—previously noted in American samples (Maltby et al. 2010; McFarland 1989), are reflected here. Because, to some extent, men and women might construe religious behavior and beliefs differently, our measure of religiosity might not have been sufficient to uncover existing associations. This issue deserves attention in future research.

The lack of significant direct and indirect links between religiosity and hostile sexism, meanwhile, is consistent with previous research and with our argumentation that religions explicitly eschew derogation of women. Also in line with previous findings (e.g., Glick et al. 2000; Mikołajczak and Pietrzak 2013), men as compared to women in our sample showed greater support both for hostile and benevolent sexist attitudes, but the difference was more pronounced in the case of hostile sexism.

Previous research has shown that in terms of values, women attribute more importance to self-transcendence (benevolence and universalism), while men espouse more openness to change and self-enhancement (Schwartz and Rubel 2005). Although, we found no differences between the genders in relative importance given to value dimensions, it is plausible that this is due to our fairly small sample size: the conclusion from Schwartz and Rubel’s (2005) metaanalysis is that effects are usually small in size, and so might be revealed only in larger samples.

Contrary to previous research (Saroglou et al. 2004; Schwartz and Huismans 1995), we found no direct link between religion and values relating to self-transcendence. Similarly, no indirect link between religiosity and sexism leading through these values was observed. This again could be due to the way in which we assessed religiosity in our study, looking at declared belief and practice, rather than more nuanced, subjective measures of religion. It could be that religious practice itself is a form of religiosity that is connected to ritual, adherence to tradition, repetition of actions—aspects we might expect to be associated with Conservatism. Meanwhile, measures focused on deeper meaning, a moral and spiritual compass, might tap more into aspects of religiosity that would more naturally cohere with Self-transcendence. Thus, more sophisticated measures of religiosity (e.g., Allport and Ross 1967; Batson et al. 1978) might serve to reconcile these findings with previous research.

Several other points of interest emerged from this study with respect to the links between ambivalent sexism and age and education, which have been equivocal so far. Results from two large community samples, one in Spain (BS positively correlated with age, negatively correlated with education; Glick et al. 2002a) and one in Israel (BS uncorrelated with age, negatively correlated with education only among men; Gaunt 2012), were inconsistent; nor were they compatible with results found in our prior research with a Polish sample (BS negatively correlated with age only among women, negatively correlated with education: Mikołajczak and Pietrzak 2013).

In the present study, age was unrelated to sexism in both genders, which corroborates results obtained by Glick and colleagues (2002a) in the case of hostile sexism, and results obtained by Gaunt (2012) and Mikołajczak and Pietrzak (2013) for benevolent sexism among men. Future studies could investigate the source of discrepancies in this variable. In both genders, age predicted greater importance attributed to conservatism over openness to change, and to self-transcendence over self-enhancement, which is in line with previous findings (Schwartz 1992).

As in the Spanish study (Glick et al. 2002a), education in our sample was negatively related to hostile sexism among men and negatively related to benevolent sexism among women (however, this relationship became insignificant when controlling for religiosity). As with age, future studies could resolve the inconsistencies observed in various samples.

### Limitations

An obvious limitation to these studies is their correlational nature, due to which definitive causal inferences about the relationship between religiosity, values, and sexism cannot be made. Based on previous findings (Hayward and Kemmelmeier 2011; Paloutzian et al. 1999) we can speculate that the directionality is likely to lead from religious engagement through values to attitudes. However, an alternative explanation, assuming that both religiosity and sexism could be perceived as manifestations of broader traditional values (cf. Inglehart and Baker 2000), might also be considered in future studies.

Although mediational models that we tested with other directionalities were not significant, experimental studies are needed to support these preliminary ideas. We conjecture that priming religiosity among believers would activate specific values that in turn would lead to greater support for benevolent sexism. Furthermore, we imagine that directly activating specific (conservative) values could itself lead to more sexist responses.

Additionally, a more refined view of values, as recently proposed by Schwartz and colleagues (2012), would allow for more specific predictions about links to attitudes. The most relevant elaboration from the point of view of these studies is the distinction between personal and societal security. While the former is a value focused on the self—personal belonging, cleanliness, avoidance of indebtedness—the latter is focused on the stability and predictability of the social system in which we function. This value is closely associated with abiding to social norms and conventions, the “rules” component of the conformity value. Disambiguating these two aspects of security might lead to new connections between these values and benevolent sexism. The finer distinction within the tradition value, separating a ‘humility’ component from a purer ‘tradition’ component, might also influence these relationships. Similarly for values of self-transcendence, a new distinction into ‘dependability’ and ‘caring’ components within benevolence values, and into ‘concern’ for all humanity and ‘tolerance’ components within universalism values (Schwartz et al. 2012) could shed more light on the link between religiosity and sexism.

Our study was also potentially limited by our lack of contextualization of religiosity. Religion is primarily transmitted intergenerationally through the family (e.g., Bengtson et al. 2009; Myers 2004). Including questions regarding family history with religion would allow for a fuller analysis of the interplay of values, attitudes, and religious belief. This is particularly relevant because of the observed intergenerational transmission of benevolent sexism (Montañés et al. 2012). Additionally, the influence of the specific national context should be examined. The proportion of the population that is religious, as well as the quality of the relationship between church and state, could influence how values translate to attitudes (Roccas 2005; Roccas and Schwartz 1997). It is plausible that in more religious nations, where the church has more political power, the values promoted by religious institutions would more strongly shape attitudes than in countries where such influence is more marginal (Roccas 2005; Schwartz 2013).

Future studies would also benefit from the inclusion of more conceptually sophisticated measures of religiosity (see, e.g., Hill 2005; Saroglou 2011). For example, it is possible that among men benevolent sexism correlates with measures more akin to intrinsic religiosity. Moreover, to the extent that intrinsically religious individuals perceive themselves as caring and compassionate toward others (self-transcendence), rather than striving for power or achievement (self-enhancement), they might be also less hostile sexist. Religious fundamentalism, on the other hand, might be linked with more support for hostile sexist attitudes. Thus, despite the predictive power of church attendance (Glick et al. 2002a; Sanchez and Hall 1999; Tarakeshwar et al. 2003), more nuanced indicators of religiosity, such as well-validated scales of intrinsic, extrinsic, quest religiosity (e.g., Batson et al. 1978), as well as fundamentalism (e.g., Altemeyer and Hunsberger 1992), would surely illuminate the ways in which religion, values, and sexism are linked.

The distribution of religiosity in our sample (see Table 1) was slightly different from what is typically obtained in representative samples in Poland (e.g., Centrum Badania Opinii Społecznej 2013a) with an overrepresentation of non-believers to the detriment of regular practitioners. For the purposes of our study, this was fortuitous—it allowed us test our hypotheses using a broader scale of experience than would be likely with a more representative (religiously homogenous) sample. On the other hand, the relationships observed in this sample might not generalize to samples with fewer non-believers. This sampling bias is likely amplified by the relatively higher education level of our sample, relative to the norm for Poland. It is likely that passengers of long-distance trains include a greater proportion of members of the middle-class or upper-middle classes than would be found in, for example, local commuter trains. Our sample is more typical of Polish society than a student sample would be, but is, nonetheless, not fully representative.

For the aforementioned reasons, the implications of the study results should be treated with due caution. We hope that future research will take some of these limitations into consideration and build on the results presented here.

### Conclusion

Our assumption that the link between Catholic religiosity and benevolent sexism might be partially explained by endorsement of more global belief constructs—values—received empirical support, complementing and extending previous studies on religiosity and sexism.

While the negative effects of hostile sexism are undisputed, there is less social acknowledgement of the negative consequences of benevolent sexism. These effects are documented in research, but remain obscured, to their perpetrators and targets alike, by the fact of their indirect influence. Thus, questions remain concerning the mechanisms of perpetuation of benevolent sexism—and the mechanisms required for its elimination. If churches and other trusted and powerful social institutions are unwittingly fostering discrimination, as indicated in our study, we can hardly expect imminent societal change. The simple conclusion from our study is that one indirect effect of promoting tradition, stability, and security is the perpetuation of an unequal status quo. The complicated follow-up: how do we respect our history and maintain social harmony while changing the world?

## Appendix

**Table 5:** Polish wording for the Ambivalent Sexism Inventory items

	English version	Polish version
1.	No matter how accomplished he is, a man is not truly complete as a person unless he has the love of a woman.	Bez względu na swoje osiągnięcia zawodowe, mężczyzna nie jest całością bez miłości kobiety.
2.	Many women are actually seeking special favors, such as hiring policies that favor them over men, under the guise of asking for “equality.”	Pod pretekstem równouprawnienia wiele kobiet zabiega o specjalne przywileje, takie jak faworyzująca polityka zatrudnienia.
3.	In a disaster, women ought to be rescued before men.	W razie katastrofy najpierw należy ratować kobiety, potem mężczyzn.
4.	Most women interpret innocent remarks or acts as being sexist.	Większość kobiet interpretuje niewinne uwagi lub zachowania jako seksistowskie.
5.	Women are too easily offended.	Kobiety zbyt łatwo się obrażają.
6.	People are not truly happy in life without being romantically involved with a member of the other sex.	Ludzie nie są naprawdę szczęśliwi, jeśli nie są w związku uczuciowym z osobą przeciwnej płci.
7.	Feminists are seeking for women to have more power than men.	Feministki dążą do tego, by kobiety miały większą władzę niż mężczyźni.
8.	Many women have a quality of purity that few men possess.	Wiele kobiet charakteryzuje się czystością moralną, rzadko spotykaną u mężczyzn.
9.	Women should be cherished and protected by men.	Kobiety powinny być wielbione i chronione przez mężczyzn.
10.	Most women fail to appreciate fully all that men do for them.	Większość kobiet nie docenia w pełni tego, co robią dla nich mężczyźni.
11.	Women seek to gain power by getting control over men.	Przejmując kontrolę nad mężczyznami, kobiety dążą do zdobycia władzy.
12.	Every man ought to have a woman whom he adores.	Każdy mężczyzna powinien mieć partnerkę, którą adoruje.
13.	Men are incomplete without women.	Mężczyzna nie jest całością bez kobiety.
14.	Women exaggerate problems they have at work.	Kobiety wyolbrzymiają problemy, które mają w pracy.
15.	Once a woman gets a man to commit to her, she usually tries to put him on a tight leash.	Z chwilą, gdy kobieta zdobędzie już mężczyznę, zazwyczaj próbuje trzymać go “krótko”.
16.	When women lose to men in a fair competition, they typically complain about being discriminated against.	Kiedy kobiety przegrywają z mężczyznami w uczciwej rywalizacji, zwykle narzekają, że są dyskryminowane.
17.	A good woman should be set on a pedestal by her man.	Dobra żona powinna być stawiana na piedestale przez swojego męża.
18.	Many women get a kick out of teasing men by seeming sexually available and then refusing male advances.	Wielu kobietom sprawia frajdę, gdy drażnią mężczyzn udając, że są dostępne seksualnie, a następnie odrzucając męskie zaloty.
19.	Women, compared to men, tend to have a superior moral sensibility.	W porównaniu z mężczyznami kobiety wydają się mieć większą wrażliwość moralną.
20.	Men should be willing to sacrifice their own wellbeing in order to provide financially for the women in their lives.	Mężczyzna powinien być gotowy poświęcić własne dobro, by zapewnić utrzymanie bliskim kobietom.
21.	Feminists are making unreasonable demands of men.	Feministki stawiają nierozsądne żądania wobec mężczyzn.
22.	Women, as compared to men, tend to have a more refined sense of culture and good taste.	W porównaniu z mężczyznami, kobiety mają bardziej wyrafinowany gust i poczucie dobrego smaku.
